# Impact of Cytokine Genes on TMD and QoL in Construction Workers

**DOI:** 10.1590/0103-644020256581

**Published:** 2026-01-09

**Authors:** André Miquilussi Moreira, Samantha Schaffer Pugsley Baratto, Débora Cristina Cardozo Bueno, Gisele Maria Correr, Cristiano Miranda de Araujo, Christian Kirschneck, Bianca Cavalcante-Leão, Erika Calvano Küchler, Flares Baratto-Filho, Michelle Nascimento Megerx

**Affiliations:** 1 School of Dentistry, Tuiuti University of Paraná, Curitiba, Paraná, Brazil; 2 School of Dentistry, UniDomBosco University Center, Curitiba, Paraná, Brazil; 3 Department of Restorative Dentistry, Federal University of Paraná, Curitiba, Paraná, Brazil; 4 School of Dentistry University of Joinville, Joinville, Santa Catarina, Brazil; 5 Department of Orthodontics, University Hospital Bonn, Medical Faculty, Welschnonnenstr. 17, 53111, Bonn, Germany

**Keywords:** Temporomandibular joint disorders, Interleukin-1 beta, Interleukin-6, Quality of life, Genetics

## Abstract

Temporomandibular disorders (TMD) include changes related to the presence of orofacial pain, limited mouth opening, and inflammatory issues. These inflammatory issues are related to interleukins, which are inflammatory mediators. Thus, the presence of genetic polymorphisms in genes that encode interleukins could impact TMD. Therefore, this cross-sectional study aimed to assess the impact of genetic polymorphisms in the genes encoding interleukins on the oral health-related quality of life (OHRQoL) of individuals with TMD. A total of 230 male construction workers were evaluated regarding the presence of TMD with or without pain, as well as changes in the temporomandibular joint (TMJ), using the Research Diagnostic Criteria for Temporomandibular Disorders (RDC/TMD). OHRQoL was assessed using the 14-item Oral Health Impact Profile (OHIP-14). DNA extracted from saliva was used to evaluate the genetic polymorphisms in *IL1B* (rs1143627 and rs1143629) and *IL6* (rs1800795 and rs1800796) using real-time polymerase chain reaction. Student's t-test or ANOVA was used for statistical analysis (alpha=5%). An association between OHIP-14 and TMD was observed (*p*<0.05). Individuals’ heterozygous AG in rs1143629 showed increased values in domains 2 (physical pain) and 4 (physical disability) compared to AA homozygotes (*p*=0.035; *p*=0.042, respectively). Individuals homozygous for the C allele at rs1800796 demonstrated higher values in domain 7 (handicap) compared to heterozygous CG and homozygous GG individuals (*p*=0.007). In conclusion, our study supported the hypothesis that TMD and genetic polymorphisms associated with inflammatory disorders are associated with OHRQoL

## Resumo

As disfunções temporomandibulares (DTM) incluem alterações relacionadas à presença de dor orofacial, limitação da abertura bucal e problemas inflamatórios. Esses problemas inflamatórios estão relacionados às interleucinas, que são mediadores inflamatórios. Assim, a presença de polimorfismos genéticos em genes que codificam interleucinas pode impactar a DTM. Portanto, este estudo transversal teve como objetivo avaliar o impacto de polimorfismos genéticos nos genes que codificam interleucinas na qualidade de vida relacionada à saúde bucal (QVRSB) de indivíduos com DTM. Um total de 230 trabalhadores da construção civil do sexo masculino foram avaliados quanto à presença de DTM com ou sem dor, bem como alterações na articulação temporomandibular (ATM) utilizando os Critérios de Diagnóstico de Pesquisa para Disfunções Temporomandibulares (RDC/TMD). A QVRSB foi avaliada utilizando o Perfil de Impacto à Saúde Bucal (OHIP-14) de 14 itens. O DNA extraído da saliva foi usado para avaliar os polimorfismos genéticos em IL1B (rs1143627 e rs1143629) e IL6 (rs1800795 e rs1800796) usando reação em cadeia da polimerase em tempo real. O teste t de Student ou ANOVA foi usado para análise estatística (alfa = 5%). Uma associação entre OHIP-14 e DTM foi observada (p < 0,05). Os indivíduos heterozigotos AG em rs1143629 apresentaram valores aumentados nos domínios 2 (dor física) e 4 (incapacidade física) em comparação com homozigotos AA (p = 0,035; p = 0,042, respectivamente). Indivíduos homozigotos para o alelo C em rs1800796 demonstraram valores mais altos no domínio 7 (deficiência) em comparação com indivíduos heterozigotos CG e homozigotos GG (p = 0,007). Em conclusão, nosso estudo apoiou a hipótese de que DTM e polimorfismos genéticos associados a distúrbios inflamatórios estão associados à qualidade de vida relacionada à saúde (QVRSB).

**Figure:**
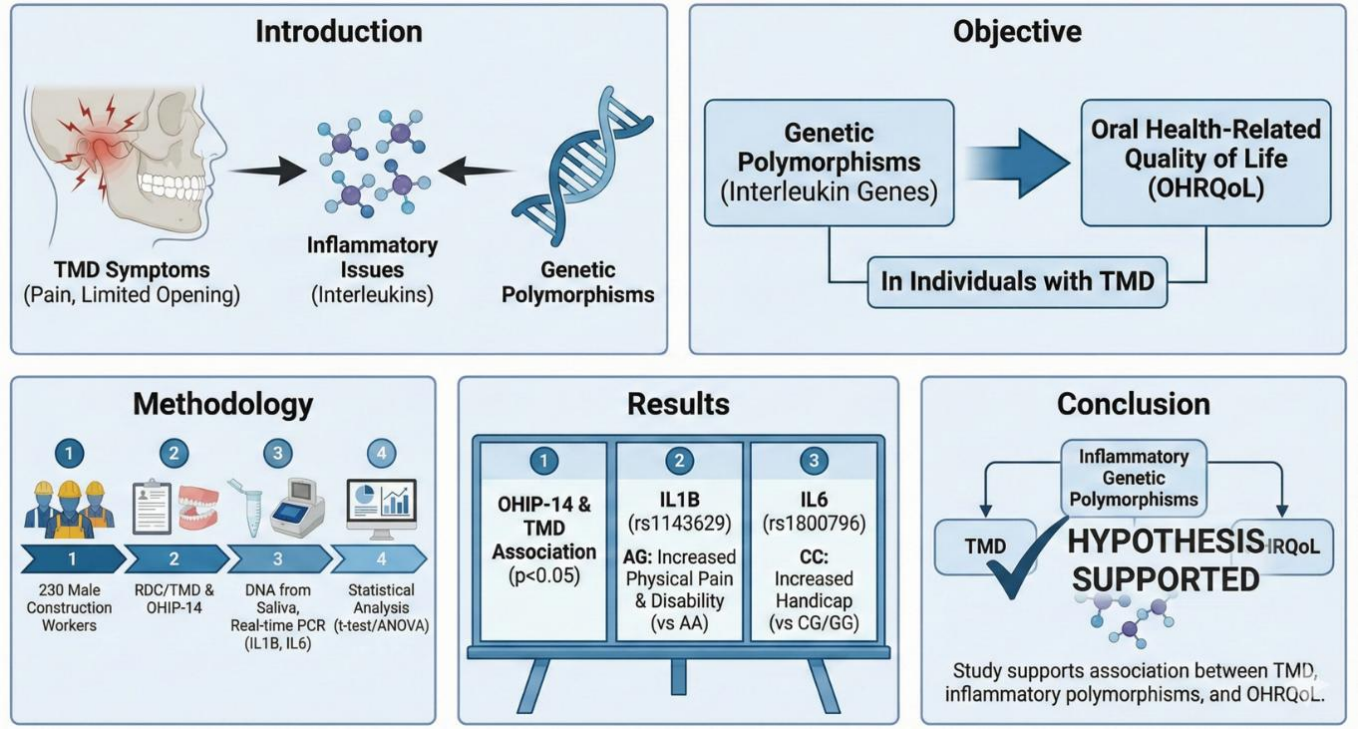

## Introduction

Temporomandibular disorders (TMDs) encompass a heterogeneous group of musculoskeletal conditions affecting the temporomandibular joint (TMJ), masticatory muscles, and associated structures. Characterized by symptoms such as orofacial pain, joint noises, and restricted jaw movement, TMDs can significantly impair essential functions such as chewing and speech ^1^ ^,^ ^2^. Consequently, these disorders often have a detrimental impact on an individual's Oral Health-Related Quality of Life (OHRQoL), affecting psychological well-being and social functioning ^3^ ^,^ ^4^ ^,^ ^5^. The etiology of TMDs is multifactorial, involving a complex interplay of biological, psychosocial, and environmental factors, including genetic predispositions ^2^.

A standardized assessment is essential for both clinical management and research consistency. To this end, the Research Diagnostic Criteria (RDC/TMD) provides a validated dual-axis system. Axis I focuses on the clinical diagnosis of physical conditions based on a standardized physical examination, while Axis II assesses psychosocial status and pain-related disability, recognizing the biopsychosocial complexity of these disorders ^3^ ^,^ ^6^.

To quantify the functional and psychosocial consequences of TMD, validated instruments are used to measure patient-reported outcomes. The Oral Health Impact Profile (OHIP) is a widely used questionnaire for this purpose. The 14-item version (OHIP-14) is a self-administered tool that efficiently assesses seven domains of OHRQoL: functional limitation, physical pain, psychological discomfort, physical disability, psychological disability, social disability, and impairment, providing a comprehensive measure of patient-perceived burden ^5^ ^,^ ^7^.

Underlying the clinical manifestations of painful TMD is a complex pathophysiology, in which inflammation is recognized as a key mechanism. In joint conditions such as disc displacement and degenerative joint disease, the local accumulation of inflammatory mediators is the primary trigger for pain ^8^. These inflammatory processes are coordinated by cytokines, particularly pro-inflammatory interleukins, which mediate cartilage and bone degradation. Specifically, elevated concentrations of interleukin-1 beta (IL-1β) and interleukin-6 (IL-6) have been identified in the synovial fluid of patients with TMD, directly implicating them in the progression of joint pathology ^9^.

Individual susceptibility to such inflammatory conditions is increasingly understood to have a genetic basis. Functional polymorphisms in genes encoding pro-inflammatory cytokines, such as IL1B and IL6, can alter mediator production and modulate the intensity of the inflammatory response, thus influencing the risk and severity of TMD ^9^. Specific populations, such as male construction workers, have genetic profiles that may predispose them to TMD symptoms, making them a relevant population for investigating these associations ^10^.

Given the interplay between inflammatory mediators, genetic predisposition, and the significant impact of TMD on daily life, this study was designed to address specific gaps in a high-risk occupational group. Therefore, the primary objective was to evaluate the impact of TMD-related pain on OHRQoL in male construction workers. The secondary objective was to investigate the association between specific genetic polymorphisms in IL1B (rs1143627 and rs1143629) and IL6 (rs1800795 and rs1800796) and the OHRQoL domains, measured by the OHIP-14, in this population.

## Materials and methods

### Ethical Considerations

This cross-sectional study was conducted at the Dental Clinic of the Social Service of the Civil Construction Industry of the State of Paraná (SECONCI-PR), located in southern Brazil. The project was approved by the Human Research Ethics Committee under protocol number 2.802.708 (CAAE: 94262618.4.0000.0093).

### Study Design

Male construction workers were recruited consecutively between 2018 and 2019. Following an initial consultation where study objectives and procedures were explained, written informed consent was obtained from all enrolled participants.

The inclusion criteria specified healthy male workers aged 18 years or older. Exclusion criteria were: ^1^ use of analgesic or anti-inflammatory medication within the preceding six months; ^2^ presence of any systemic comorbidities; and ^3^ illiteracy, which precluded the ability to provide valid written consent. The design and reporting of this study adhere to the STROBE (Strengthening the Reporting of Observational Studies in Epidemiology) statement and its STREGA extension for genetic association studies ^11^.

### Clinical Variables

All clinical examinations were performed by a single, senior dentist, certified and experienced in the diagnosis of temporomandibular disorders (TMDs). Participants were diagnosed according to the Axis I physical assessment protocol of the Research Diagnostic Criteria for Temporomandibular Disorders (RDC/TMD).

The Axis I examination protocol included palpation of the masticatory muscles and temporomandibular joints using standardized pressures of 1.0 kg and 0.5 kg, respectively. Mandibular range of motion and the presence of pain during jaw function were also assessed. Based on these findings, participants were classified into diagnostic subgroups. Muscle dysfunctions were categorized as myofascial pain, with or without limitation of mouth opening. Joint dysfunctions were assessed bilaterally and classified as arthralgia, defined as joint pain on palpation, or osteoarthritis, identified by the presence of joint crepitus during function in the absence of concomitant pain.

### Evaluation of OHRQoL

OHRQoL was assessed using the OHIP-14 questionnaire. This instrument measures seven domains: functional limitation, physical pain, psychological discomfort, physical disability, psychological disability, social disability, and handicap. Responses were coded on a five-point Likert scale (0 = never, 1 = rarely, 2 = sometimes, 3 = often, 4 = always). The score for each domain, ranging from 0 to 8, was calculated by summing the scores of the two corresponding items. The total OHIP-14 score, obtained by the summation of all domain scores, ranged from 0 to 56, with higher scores indicating poorer OHRQoL

### Genetic Analysis

For genetic analysis, saliva was collected from all participants in 15 mL Falcon tubes and stored at -20°C. Genomic DNA extraction from saliva was performed as previously described ^12^. Genetic polymorphisms in *IL1B* (rs1143627 and rs1143629) and *IL6* (rs1800795 and rs1800796) were investigated using real-time polymerase chain reaction (RT-PCR) on the StepOnePlus Real-time PCR System (Applied Biosystems, USA) with the TaqMan assay (Applied Biosystems, Foster City, CA, USA). Table 1 presents the candidate genes and genetic polymorphisms.

For reactions, a total volume of 3 μL (1.425 μL of water, 1.5 μL TaqMan PCR master mix, 0.075 μL SNP assay; Applied Biosystems, Foster City, CA) and 4ng of DNA per sample was used. The thermal profile was configured as follows: pre-incubation cycle at 95°C (10 min) and 40 amplification cycles at 92°C (15 s) and 60°C (1 min). Two negative controls were included on each plate. An internal consistency test was conducted by randomly reprocessing 10% of all samples, resulting in 100% consistency.

### Statistical analysis

The data was analyzed as a continuous variable, and normality was tested using the Kolmogorov-Smirnov test. As the data followed a normal distribution, the Student's t-test was used to compare inflammatory disorders with OHIP-14 domain scores when there were two groups, while one-way analysis of variance (ANOVA) was employed when there were three groups. ANOVA was also used to investigate variations in the mean scores of each OHIP-14 domain, genotypes, and phenotypes, such as myofascial pain and inflammatory disorders. When ANOVA indicated a significant difference, the Tukey post-hoc test was conducted for pairwise comparisons. All analyses were performed using the statistical software Jasp, version 0.14.1, with a significance level set at 5%.

A test for deviation from the Hardy-Weinberg equilibrium was performed with the chi-square test. The post-hoc power calculator was performed using the clinclac.com.

**Table 1:** Candidate genes e polymorphisms.

Gene	Genetic polymorphism	Position	Type of alteration	Mutant allele	MAF	Genotypes n(%)			**H-W *p-*value**
						Homozygous dominant	Heterozygous	Homozygous recessive	
*IL1B* (*Interleukin 1 beta*)	rs1143627	Chromosome 2: 112836810 (GRCh38)	Transcript Variant	G/A	0.47	68 (33.3)	85 (41.7)	51 (25.0)	0.021
	rs1143629	Chromosome 2: 112835941 (GRCh38)	Intron Variant	A/G	0.48	77 (37)	77 (37)	54 (26)	0.0003
*IL6* (*Interleukin 6*)	rs1800795	Chromosome 7:22727026 (GRCh38)	Intron Variant	C/G	0.14	114 (53.0)	78 (36.3)	23 (10.7)	0.089
	rs1800796	Chromosome 7: 22726627 (GRCh38)	Transcript Variant	C/G	0.31	131 (63.6)	67 (32.5)	8 (3.9)	0.877

Obtained from database: https://www.ncbi.nlm.nih.gov/snp/

*MAF* minor allele frequency

H-W Hardy-Weinberg

## Results

A total of 230 male individuals were included in the study. Ages ranged from 18 to 77 years, and the mean age was 37.8 ± 11.0 years. A total of 31 individuals reported bruxism, and 36 reported clenching.

Linear regression analysis was not performed, as the study's focus was on comparing mean differences across groups using ANOVA, rather than modeling continuous associations. Table 2(a and b) shows the variables studied and the OHIP-14 domains. A significant association between the OHIP-14 (total scale and all domains) and the TMD variables was observed (*p* < 0.05). An association between the presence of pain and the domains of the total scale, physical pain, psychological discomfort, physical, psychological, and social disability, and handicap (*p* < 0.001; *p* = 0.028; *p* = 0.005; *p* = 0.045; *p* < 0.001; *p* = 0.007; *p* = 0.034, respectively) was also observed. The presence of inflammatory disorders was also associated with the domain of total scale, physical pain, psychological discomfort, psychological and social disability (*p* = 0.001; *p* = 0.044; *p* = 0.001; *p* = 0.013; *p* = 0.002, respectively). Right and left side inflammatory disorders were classified into inflammatory absence, arthralgia, osteoarthritis, and osteoarthrosis. On the right side, joint pain, arthralgia, was associated with the domain of total scale, psychological discomfort, and social disability (*p* = 0.003; *p* = 0.004; *p* = 0.001, respectively). On the left side, there was an association between the absence of inflammation and arthralgia in the domain of total scale, functional limitation, psychological and social disability (*p* = 0.008; *p* = 0.044; *p* = 0.024; *p* = 0.019, respectively). In the psychological discomfort domain, there was an association between the absence of inflammation and osteoarthritis (*p* = 0.013).

The association between OHIP-14 scores and genetic polymorphisms in *IL1B* (rs1143627 and rs1143629) and *IL6* (rs1800795 and rs1800796) is summarized in Tables 3 and 4, respectively. In Table 3, individuals’ heterozygous AG in rs1143629 showed increased values in domains 2 (physical pain) and 4 (physical disability) compared to AA homozygotes (*p* = 0.035; *p* = 0.042, respectively). It was also possible to observe an association in Table 4 between individuals homozygous for the C allele at rs1800796, who demonstrated higher values in domain 7 (handicap) compared to heterozygous CG and homozygous GG individuals (*p* = 0.007).

The post-hoc power ranged from 60% to 3%.

**Table 2a:** Comparison of OHIP-14 scores between myofascial pain, inflammatory disorder, and right and left inflammatory disorder variables

	OHIP-14							
	Total Scale		Functional limitation		Physical pain		Psychological discomfort	
Variables	Mean (SD)	*p* value	Mean (SD)	*p* value	Mean (SD)	*p* value	Mean (SD)	*p* value
Myofascial Pain								
Absence	6.39 (6.11)^a^	< 0.001*	0.27 (0.93)^a^	0.692	0.91 (1.44)^a^	0.028*	1.72 (1.91)^a^	0.005*
Pain	12.50 (7.01)^b^		0.44 (1.04)^a^		1.88 (1.81)^b^		3.00 (2.14)^b^	
Pain with limitation	11.00 (1.41)^ab^		-		1.00 (1.41)^ab^		4.50 (0.70)^ab^	
Inflammatory disorder								
Absence	6.14 (6.26)	0.001*	0.27 (0.96)	0.723	0.87 (1.45)	0.044*	1.61 (1.91)	0.001*
Presence	9.28 (6.19)		0.32 (0.87)		1.33 (1.56)		2.58 (1.94)	
Right inflammatory disorder								
Absence	6.15 (6.19)^a^	0.003*	0.30 (1.00)^a^	0.339	0.88 (1.44)^a^	0.070	1.61 (1.90)^a^	0.004*
Arthralgia	11.44 (7.81)^b^		0.50 (1.04)^a^		1.33 (1.41)^a^		2.94 (2.15)^b^	
Osteoarthrosis	8.21 (6.16)^ab^		0.05 (0.22)^a^		1.05 (1.50)^a^		2.31 (1.76)^ab^	
Osteoarthritis	9.15 (3.57)^ab^		-		1.92 (1.93)^a^		2.92 (1.93)^ab^	
Left inflammatory disorder								
Absence	6.30 (6.19)^a^	0.008*	0.26 (0.93)^a^	0.044*	0.95 (1.50)^a^	0.512	1.65 (1.89)^a^	0.013*
Arthralgia	11.78 (8.52)^b^		0.92 (1.49)^b^		1.35 (1.39)^a^		2.57 (2.65)^ab^	
Osteoarthrosis	7.85 (5.91)^ab^		0.14 (0.47)^ab^		0.85 (1.38)^a^		2.38 (1.74)^ab^	
Osteoarthritis	9.20 (3.88)^ab^		-		1.50 (1.65)^a^		3.30 (1.82)^b^	

**Table 2b:** Comparison of OHIP-14 scores between myofascial pain, inflammatory disorder, and right and left inflammatory disorder variables

	OHIP-14							
	Physical disability		Psychological disability		Social disability		Handicap	
Variables	Mean (SD)	*p* value	Mean (SD)	*p* value	Mean (SD)	*p* value	Mean (SD)	*p* value
Myofascial Pain								
Absence	0.96 (1.84)^a^	0.045*	1.43 (1.54)^a^	< 0.001*	0.72 (1.01)^a^	0.007*	0.38 (0.98)^a^	0.034*
Pain	2.11 (2.37)^b^		2.83 (1.29)^b^		1.38 (0.91)^b^		1.05 (1.58)^b^	
Pain with limitation	0.50 (0.70)^ab^		2.50 (0.70)^ab^		2.00 (0.00)^ab^		0.50 (0.70)^ab^	
Inflammatory disorder								
Absence	0.94 (1.84)	0.124	1.40 (1.56)	0.013*	0.67 (0.99)	0.002*	0.39 (1.00)	0.279
Presence	1.39 (2.05)		2.00 (1.50)		1.16 (1.02)		0.57 (1.17)	
Right inflammatory disorder								
Absence	0.94 (1.83)^a^	0.123	1.40 (1.56)^a^	0.056	0.66 (0.98)^a^	0.001*	0.40 (1.00)^a^	0.288
Arthralgia	2.05 (2.31)^a^		2.22 (1.76)^a^		1.50 (0.85)^b^		0.88 (1.53)^a^	
Osteoarthrosis	1.21 (2.29)^a^		2.00 (1.41)^a^		1.10 (1.28)^ab^		0.47 (1.17)^a^	
Osteoarthritis	0.92 (1.44)^a^		2.00 (1.15)^a^		1.15 (0.80)^ab^		0.30 (0.63)^a^	
Left inflammatory disorder								
Absence	0.98 (1.85)^a^	0.142	1.40 (1.54)^a^	0.024*	0.69 (0.98)^a^	0.019*	0.38 (0.98)^a^	0.095
Arthralgia	2.14 (2.38)^a^		2.35 (1.86)^b^		1.35 (1.08)^b^		1.07 (1.68)^a^	
Osteoarthrosis	1.14 (2.19)^a^		1.90 (1.48)^ab^		1.04 (1.20)^ab^		0.57 (1.16)^a^	
Osteoarthritis	0.60 (0.84)^a^		2.40 (0.96)^ab^		1.30 (0.82)^ab^		0.20 (0.42)^a^	

**Table 3:** Comparison of OHIP-14 scores with *IL1B* polymorphisms (rs1143627 and rs1143629)

OHIP-14	IL1B polymorphisms / genotypes		Mean (SD)	***p*-value**
Total scale	rs1143627	AA	5.58 (± 5.12)	0.071
		GA	7.84 (± 7.00)	
		GG	7.82 (± 7.37)	
	rs1143629	AA	5.92 (± 5.40)	0.118
		AG	6.89 (± 6.16)	
		GG	8.31 (± 8.13)	
Functional limitation	rs1143627	AA	0.27 (± 1.02)	0.592
		GA	0.38 (± 1.11)	
		GG	0.21 (± 0.70)	
	rs1143629	AA	0.32 (± 1.00)	0.969
		AG	0.28 (± 1.05)	
		GG	0.29 (± 0.86)	
Physical pain	rs1143627	AA	0.70 (± 1.13)	0.064
		GA	1.05 (± 1.59)	
		GG	1.35 (± 1.73)	
	rs1143629	AA	0.72 (± 1.13)a	0.035*
		AG	0.94 (± 1.58)ab	
		GG	1.40 (± 1.72)b	
Psychological discomfort	rs1143627	AA	1.63 (± 1.76)	0.393
		GA	2.07 (± 2.12)	
		GG	1.80 (± 2.05)	
	rs1143629	AA	1.71 (± 1.73)	0.536
		AG	2.03 (± 1.98)	
		GG	1.74 (± 2.20)	
Physical disability	rs1143627	AA	0.72 (± 1.48)	0.154
		GA	1.24 (± 2.14)	
		GG	1.33 (± 2.14)	
	rs1143629	AA	0.83 (± 1.59)a	0.042*
		AG	0.89 (± 1.84)ab	
		GG	1.63 (± 2.37)b	
Psychological disability	rs1143627	AA	1.29 (± 1.27)	0.205
		GA	1.69 (± 1.66)	
		GG	1.74 (± 1.82)	
	rs1143629	AA	1.29 (± 1.28)	0.160
		AG	1.54 (± 1.54)	
		GG	1.83 (± 1.93)	
Social disability	rs1143627	AA	0.72 (± 0.87)	0.314
		GA	0.96 (± 1.08)	
		GG	0.76 (± 1.19)	
	rs1143629	AA	0.72 (± 0.91)	0.621
		AG	0.88 (± 1.01)	
		GG	0.75 (± 1.21)	
Handicap	rs1143627	AA	0.30 (± 0.83)	0.310
		GA	0.49 (± 1.19)	
		GG	0.60 (± 1.18)	
	rs1143629	AA	0.36 (± 0.88)	0.159
		AG	0.39 (± 1.01)	
		GG	0.70 (± 1.36)	

Note: ANOVA and Tukey’s post hoc test were used.

**Table 4:** Comparison of OHIP-14 scores with *IL6* polymorphisms (rs1800795 and rs1800796)

OHIP-14	IL6 polymorphisms / genotypes		Mean (SD)	***p*-value**
Total scale	rs1800795	CC	7.04 (± 4.83)	0.777
		CG	6.53 (± 5.86)	
		GG	7.21 (± 7.10)	
	rs1800796	CC	11.00 (± 9.81)	0.198
		CG	6.71 (± 6.56)	
		GG	6.76 (± 6.28)	
Functional limitation	rs1800795	CC	0.04 (± 0.20)	0.404
		CG	0.29 (± 0.79)	
		GG	0.34 (± 1.15)	
	rs1800796	CC	0.75 (± 1.75)	0.358
		CG	0.23 (± 0.79)	
		GG	0.28 (± 0.96)	
Physical pain	rs1800795	CC	1.17 (± 1.55)	0.640
		CG	0.87 (± 1.44)	
		GG	1.02 (± 1.52)	
	rs1800796	CC	2.00 (± 1.85)	0.081
		CG	1.11 (± 1.78)	
		GG	0.86 (± 1.29)	
Psychological discomfort	rs1800795	CC	1.78 (± 2.25)	0.939
		CG	1.84 (± 1.87)	
		GG	1.92 (± 2.00)	
	rs1800796	CC	0.75 (± 0.88)	0.058
		CG	1.52 (± 1.72)	
		GG	2.05 (± 2.11)	
Physical disability	rs1800795	CC	1.34 (± 1.79)	0.668
		CG	0.94 (± 1.77)	
		GG	1.09 (± 2.04)	
	rs1800796	CC	2.37 (± 3.11)	0.079
		CG	1.22 (± 2.16)	
		GG	0.89 (± 1.72)	
Psychological disability	rs1800795	CC	1.73 (± 1.42)	0.451
		CG	1.35 (± 1.52)	
		GG	1.60 (± 1.62)	
	rs1800796	CC	2.37 (± 1.92)	0.298
		CG	1.44 (± 1.50)	
		GG	1.55 (± 1.61)	
Social disability	rs1800795	CC	0.52 (± 0.79)	0.411
		CG	0.83 (± 0.98)	
		GG	0.82 (± 1.11)	
	rs1800796	CC	1.12 (± 1.88)	0.628
		CG	0.85 (± 1.07)	
		GG	0.77 (± 0.97)	
Handicap	rs1800795	CC	0.60 (± 0.94)	0.669
		CG	0.38 (± 0.91)	
		GG	0.45 (± 1.17)	
	rs1800796	CC	1.62 (± 1.84)a	0.007*
		CG	0.40 (± 1.01)b	
		GG	0.40 (± 1.02)c	

Note: ANOVA and Tukey’s post hoc test were used.

## Discussion

OHRQoL refers to the impact on the QoL related to individuals' oral health and includes physical, social, and psychological aspects that affect individuals' daily activities ^13^. In the sample of this study, the presence of TMD, especially related to pain, was shown to impact the individuals' OHRQoL. Previous studies have also shown that the experience of pain and discomfort can affect mood, sleep quality, and the ability to perform daily activities ^14^ ^,^ ^15^ ^,^ ^16^.

Most TMD patients experience chronic pain conditions that affect daily activities, including eating, chewing, and speaking. This aspect can result in compromised social behavior and psychological health. Among the subgroups of TMD, disorders of muscular origin and inflammatory and degenerative disorders of joint origin may be related to the presence of pain and also limited function, which can significantly affect the QoL of this group of patients. Two systematic reviews regarding QoL and TMD demonstrate that individuals with TMD have a negative impact on OHRQoL ^4^ ^,^ ^17^.

The questionnaire used in this research, OHIP-14, evaluates seven dimensions (functional limitation, physical pain, psychological discomfort, physical disability, psychological disability, social disability, and handicap) ^18^. The measuring instrument can measure the perception of the impact that an oral health problem can have on an individual's health ^13^. In this study, using the OHIP-14 to measure the impact on OHRQoL, the presence of myofascial pain was related to higher averages in the total index of this questionnaire and is statistically significant when compared to the group with no pain, representing a greater impact on QoL and agreeing with several studies that evaluated myofascial pain ^13^ ^,^ ^14^ ^,^ ^15^ ^,^ ^16^ ^,^ ^19^.

In relation to the OHIP-14 domains, the presence of pain had a higher mean and statistically significant impact on OHRQoL related to the presence of myofascial pain in the domains physical pain, psychological discomfort, physical disability, psychological disability, social disability and handicap, these results which agreed with a study carried out on the impact on QoL related to the TMD subgroup ^14^ ^,^ ^20^ ^,^ ^21^.

The presence of joint inflammatory disorder impacted QoL indices, denoting higher values in relation to the domains of physical pain, psychological discomfort, psychological disability, social disability, and total score. This scenario reflects the impact of pain on QoL, since inflammatory changes must be accompanied by pain. These results can be seen in other studies that evaluated the presence of joint inflammatory changes and QoL indices ^14^ ^,^ ^5^ ^,^ ^20^ ^,^ ^21^.

The investigation into OHRQoL and genetic polymorphisms is still limited. Several studies have described associations between genetic polymorphisms and quality of life (QoL), indicating that variations in DNA could act as biomarkers for QoL. Studies have shown that genetic polymorphisms in genes related to cytokines and inflammation are associated with symptoms of physical well-being, pain, fatigue, and social disadvantage ^22^ ^,^ ^23^ ^,^ ^24^ ^,^ ^25^ ^,^ ^26^ ^,^ ^27^. In this context, in this study, we explore the hypothesis that construction workers may have their OHRQoL affected.

According to the GENEQoL Consortium, the initiative to investigate potential biological pathways, genes, and genetic variants involved in OHRQoL has produced reviews on the biological and genetic mechanisms associated with symptoms that are related to OHRQoL ^28^. In our study, we observed statistical associations between the genetic polymorphism in *IL1B* (rs1143629) and the physical pain and physical disadvantage domains, and the statistical associations between the genetic polymorphism in *IL6* (rs1800796) and the handicap domain. Our findings corroborate the findings of other previous studies that evaluated QoL and genetic polymorphisms in individuals with lung cancer ^23^ ^,^ ^24^ ^,^ ^25^, patients with dentofacial deformity ^5^, and dental caries ^22^. A previous study observed that the polymorphism rs1800795 in *the IL-6* gene was associated with gingivitis, and the rs1143627 and rs1143629 in *IL-1B*were associated with dental caries and gingivitis ^29^. Interestingly, the study from Ersig et al. (2017) observed that the rs1143629 was associated with observed distress in children undergoing a medical procedure ^30^.

The present study has characteristics that should be discussed here. The first point to be highlighted is the fact that the sample was made up only of male construction workers. We know that TMDs affect women more when compared to men in a ratio of 2:1; however, although this condition affects more females, the nature of this condition in males should also be studied. Another important aspect to be mentioned is ethnic factors, as the sample is composed only of Brazilians. One important limitation is that by investigating only two genetic polymorphisms per gene, the study may have missed other functional or causative variants located elsewhere in the gene or in regulatory regions that could influence the phenotype. Therefore, to ensure the robustness of the results presented, future studies in other populations must be tested.

## Conclusion

Our study supported the hypothesis that TMD is associated with a negative impact on OHRQoL, demonstrating that men are also affected and may have limitations related to QoL when they present with myofascial pain and inflammatory disorders. The genetic polymorphisms rs1143629 in *IL1B* and rs1800796 in *IL6* were associated with the domains of physical pain, physical disability, and handicap.
